# Addressing Acromegaly-Related Malocclusion With Surgery-First Orthognathic Surgery: A Clinical Case Report

**DOI:** 10.7759/cureus.61999

**Published:** 2024-06-09

**Authors:** Flávia Pereira, Mariana Cebotari, Inês Camelo, Lígia Coelho

**Affiliations:** 1 Maxillofacial Surgery Department, Centro Hospitalar Universitário de São João, Porto, PRT

**Keywords:** malocclusion, pituitary adenoma, orthognathic surgery, acromegaly, prognathism

## Abstract

Angle’s class III malocclusions are characterized by the anterior positioning of the mandible in relation to the maxilla. The discrepancy can be caused by an anterior deficiency of the maxilla, excessive mandibular prognathism, or a combination of both.

Acromegaly is a dysfunction caused by the excessive production of growth hormone (GH), which leads to systemic changes and orofacial manifestations. In acromegaly caused by a pituitary adenoma, which secretes an excessive amount of GH, disproportionate mandibular growth may occur, leading to skeletal class III malocclusion in adulthood. Excessive growth stops when the tumor is removed, but the skeletal deformity persists, requiring orthognathic surgery to reposition the mandible.

This article reports the case of a 31-year-old man referred to the maxillofacial surgery consultation due to severe Angle’s class III malocclusion, with prognathism, mandibular asymmetry, and maxillary retrusion. He had a history of disproportionate soft tissue growth (hands and feet) up to 18 years old, less evident after that age. Considering the possibility of acromegaly due to a pituitary adenoma, imaging studies (CT scan and magnetic resonance imaging (MRI)) and directed analytical studies were requested. When the diagnosis was confirmed, the patient was referred to endocrinology and neurosurgery consultations. After undergoing endoscopic resection of the pituitary adenoma, the patient underwent surgery-first orthognathic surgery to correct the dental malocclusion.

## Introduction

Orthognathic surgery is a crucial intervention in the management of conditions such as mandibular prognathism and acromegaly. Mandibular prognathism, characterized by a protruding lower jaw, is a severe maxillofacial deformity [1]. Orthognathic procedures like intraoral vertico-sagittal ramus osteotomy are effective in addressing mandibular prognathism, particularly in cases with excessive ramus flaring and temporomandibular joint dysfunction [2]. Bimaxillary surgery has become increasingly popular for treating mandibular prognathism due to its ability to provide more favorable corrections of facial proportions and improved esthetic outcomes with enhanced stability [3].

Acromegaly, a disorder caused by excessive growth hormone (GH) production, often presents with mandibular prognathism due to jaw overgrowth, particularly affecting the ramus [4]. The association between hypertrophic frontal sinuses and mandibular overgrowth in acromegaly underscores the complex relationship between facial structures in these conditions [5].

Orthognathic surgery not only corrects jaw deformities but also influences ventilation during sleep, potentially impacting conditions like obstructive sleep apnea [6,7]. Furthermore, orthognathic surgery has been demonstrated to induce changes in stomatognathic function in patients with mandibular prognathism, highlighting the functional improvements achieved through surgical intervention [8]. Factors such as muscle balance and facial expressions play a role in post-surgical stability and esthetic outcomes, emphasizing the comprehensive approach necessary in orthognathic treatment [9].

Additionally, the long-term stability of occlusion achieved through orthognathic surgery in acromegaly cases highlights the enduring benefits of surgical intervention in managing these complex conditions [10].

## Case presentation

**Figure 1:**
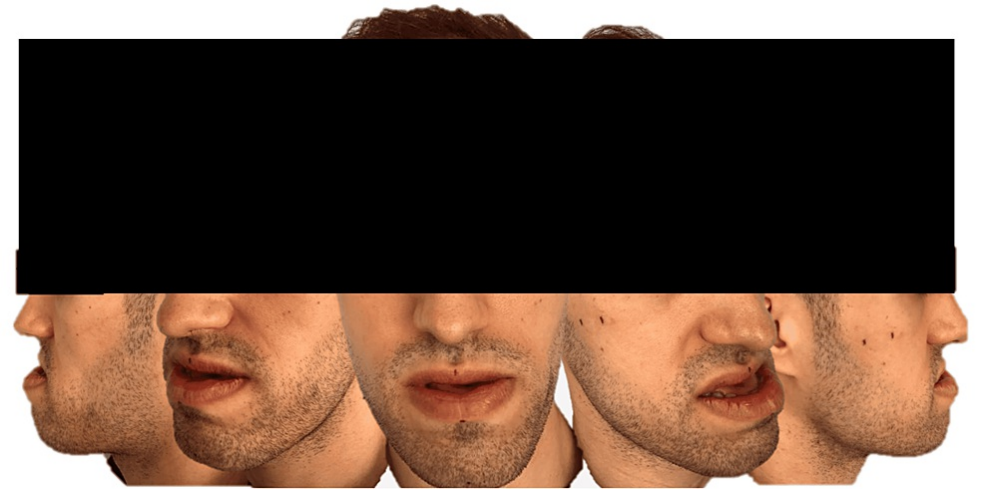
Initial pictures of the patient.

A 31-year-old man was referred to the Maxillofacial Surgery consultation for evaluation and management of a severe Angle’s class III malocclusion, with promandibula, mandibular asymmetry, and maxillary retrusion (Figure 1).

The patient’s medical history was notable for disproportionate growth of soft tissues, particularly in the hands and feet, which had been prominent until the age of 18 and had become less noticeable thereafter.

Given the clinical presentation, there was a high suspicion of acromegaly, a disorder often caused by a pituitary adenoma that secretes excessive amounts of GH, leading to abnormal skeletal growth. To confirm the diagnosis, comprehensive imaging studies, including a CT scan and magnetic resonance imaging (MRI), were conducted alongside a targeted analytical study to assess hormone levels.

The imaging and laboratory results confirmed the diagnosis of acromegaly due to a pituitary adenoma. Following this confirmation, the patient was promptly referred to the endocrinology and neurosurgery departments for further evaluation and management. The multidisciplinary team decided that the best course of action would be to proceed with endoscopic resection of the pituitary adenoma. This minimally invasive surgical approach aimed to remove the tumor, thereby halting the excessive secretion of GH and preventing further abnormal growth.

Postoperatively, the patient showed no signs of ongoing hormone overproduction, indicating a successful resection of the adenoma.

**Figure 2:**
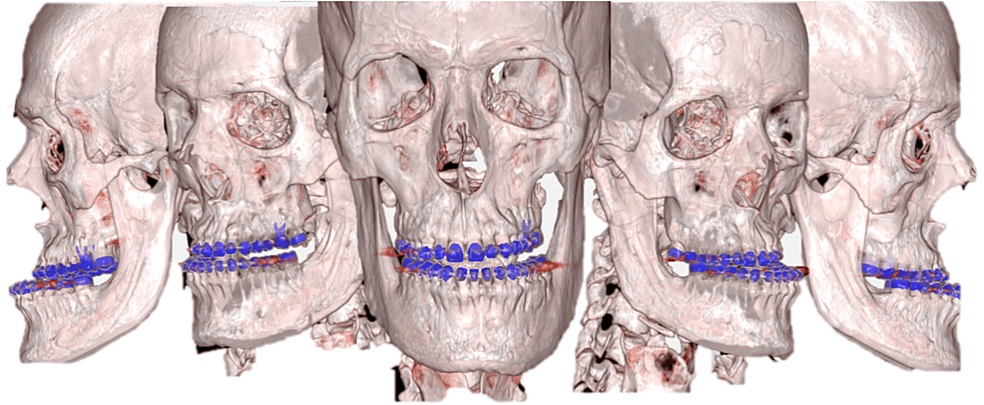
Initial CT scan with tridimensional reconstruction.

With the endocrine aspect of his condition under control, attention turned back to the severe dental and skeletal deformities resulting from years of unchecked GH effects. The next step in his treatment plan was to address the skeletal deformities through orthognathic surgery (Figure 2).

**Figure 3:**
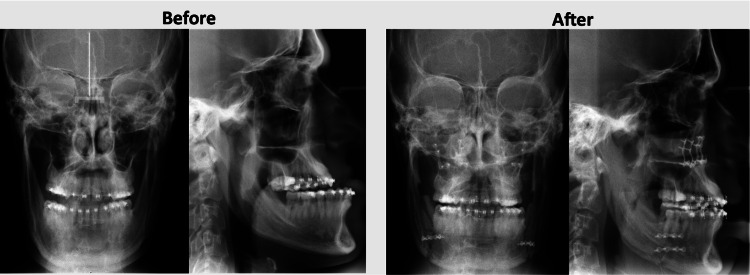
Face and profile teleradiographies (before and after orthognathic surgery).

To correct facial skeletal changes, the technique used is orthognathic surgery, using the conventional approach, with previous orthodontic correction, or the surgery-first method. In this case, the patient underwent surgery-first orthognathic surgery to correct the dental malocclusion and realign the jaw structures. This complex surgical intervention involved meticulous planning and coordination to ensure both functional and esthetic outcomes. The surgical team aimed to reposition the mandible and maxilla to achieve a balanced facial profile and proper occlusion (Figure 3).

**Figure 4:**
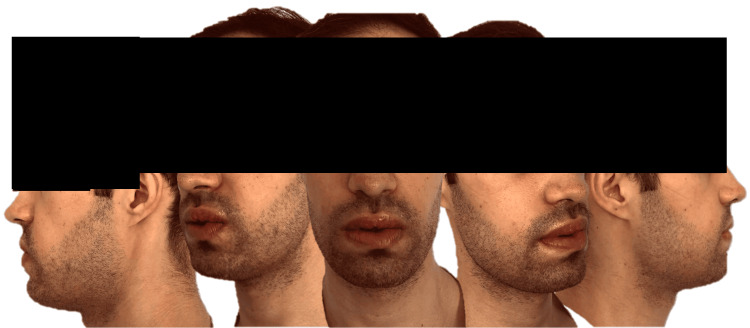
One month after surgery.

One month after surgery, the patient was doing well, free of any complaints, and expressed great satisfaction with the outcome (Figure 4).

## Discussion

Acromegaly, a rare endocrine disorder, is primarily caused by the overproduction of GH due to a GH-secreting pituitary adenoma [11]. This hormonal imbalance leads to a multitude of systemic changes and pronounced orofacial manifestations, resulting in characteristic physical manifestations such as prognathism, macroglossia, and malocclusion [12]. The insidious onset and slow progression of acromegaly often lead to delayed diagnosis, with patients typically being diagnosed five to more than 10 years after the onset of the condition [13]. The diagnosis of acromegaly is confirmed through endocrine evaluation, which includes assessing GH and insulin-like growth factor 1 (IGF-1) levels, along with imaging studies like MRI [14].

Patients with acromegaly commonly present with a range of clinical features, including soft tissue swelling, thickened nails, deepening of facial creases, enlarged pores, hypertrichosis, and hypertension [15]. Additionally, acromegaly is associated with systemic manifestations such as arthropathy, carpal tunnel syndrome, and sleep apnea. The physical disfigurement seen in acromegaly mainly affects the face and extremities, with characteristic features like coarsening of facial features and bony proliferation. Furthermore, patients with acromegaly may exhibit acral enlargement, prognathism, jaw malocclusion, and visceromegaly [16,17].

Surgical management of acromegaly involves the resection of the GH-secreting pituitary adenoma, which can lead to hormonal control and improvement in the physical manifestations of the condition [18]. Preoperative treatment with somatostatin analogs like lanreotide has been shown to increase short-term postoperative cure rates in acromegalic patients with macroadenomas [16]. 

Malocclusion, a misalignment of the teeth and jaws, is a common consequence of acromegaly due to excessive GH production affecting the craniofacial structures [18]. Among these manifestations, one of the most significant is the development of severe prognathism, particularly noted in patients with Angle’s class III malocclusions. Angle’s class III malocclusions are characterized by the anterior positioning of the mandible in relation to the maxilla. This discrepancy can arise from an anterior deficiency of the maxilla, excessive mandibular prognathism, or a combination of both factors.

Patients with acromegaly may experience mandibular prognathism, macrognathism, and alterations in jaw growth, leading to skeletal malocclusion. While the excessive growth halts following the removal of the pituitary adenoma, the skeletal deformity remains persistent, necessitating surgical intervention to reposition the mandible. Orthognathic surgery is often recommended for patients with acromegaly to address skeletal discrepancies and improve facial esthetics and function [19].

The challenges in managing late-developing malocclusions in adulthood, particularly in cases attributed to acromegaly, highlight the importance of a multidisciplinary approach involving orthodontic and surgical interventions [20]. The role of the maxillofacial surgeon is pivotal in the clinical evaluation and management of patients presenting with severe prognathism, which can often be a manifestation of acromegaly. The definitive treatment for correcting these facial skeletal changes is orthognathic surgery. This procedure can be performed using the conventional approach, which includes preoperative orthodontic correction or the surgery-first method. The selection of the appropriate surgical technique is crucial in achieving the desired outcomes.

The presented case underscores the importance of a multidisciplinary approach in managing conditions like acromegaly. Effective management requires the coordinated efforts of endocrinologists, neurosurgeons, and maxillofacial surgeons. Each specialist brings essential expertise to the table: endocrinologists manage the hormonal imbalance, neurosurgeons address the pituitary adenoma, and maxillofacial surgeons correct the resultant skeletal deformities.

A multidisciplinary approach ensures that all aspects of the patient's condition are thoroughly evaluated and treated. Endocrinologists are vital in diagnosing acromegaly and managing the hormonal imbalance. Neurosurgeons perform the crucial task of removing the pituitary adenoma, typically through endoscopic resection, which halts the excessive secretion of GH. Subsequently, the focus shifts to the maxillofacial surgeon, who undertakes the complex task of orthognathic surgery to correct the skeletal deformities caused by years of abnormal GH activity.

The comprehensive treatment plan is essential not only for resolving the immediate health issues posed by the adenoma but also for addressing the significant facial and skeletal changes that affect the patient's quality of life. The long-term structural corrections achieved through orthognathic surgery help restore functional occlusion, improve facial esthetics, and enhance overall health outcomes.

## Conclusions

In conclusion, managing skeletal Angle's class III malocclusion associated with acromegaly requires a well-coordinated, multidisciplinary approach. This case exemplifies the importance of such an approach, demonstrating that addressing both the hormonal imbalance and the resultant skeletal deformities can significantly improve patient outcomes. The integration of endocrinological, neurosurgical, and maxillofacial expertise is crucial in providing holistic care. Orthognathic surgery is a vital component in the management of acromegaly-related malocclusion, aiming to correct skeletal discrepancies and improve facial esthetics and function. Preoperative evaluation, hormonal control, and multidisciplinary collaboration are essential in optimizing the outcomes of orthognathic surgery for patients with acromegaly.
